# Paternal Care Decreases Foraging Activity and Body Condition, but Does Not Impose Survival Costs to Caring Males in a Neotropical Arachnid

**DOI:** 10.1371/journal.pone.0046701

**Published:** 2012-10-10

**Authors:** Gustavo S. Requena, Bruno A. Buzatto, Eduardo G. Martins, Glauco Machado

**Affiliations:** 1 Departamento de Ecologia, Instituto de Biociências, Universidade de São Paulo, São Paulo, SP, Brazil; 2 Centre for Evolutionary Biology, School of Animal Biology, University of Western Australia - Crawley, WA, Australia; 3 Institute of Environmental Science and Department of Biology, Carleton University, Ottawa, ON, Canada; 4 Centre for Applied Conservation Research, Department of Forest Sciences, University of British Columbia, Vancouver, BC, Canada; University of Bristol, United Kingdom

## Abstract

Exclusive paternal care is the rarest form of parental investment in nature and theory predicts that the maintenance of this behavior depends on the balance between costs and benefits to males. Our goal was to assess costs of paternal care in the harvestman *Iporangaia pustulosa*, for which the benefits of this behavior in terms of egg survival have already been demonstrated. We evaluated energetic costs and mortality risks associated to paternal egg-guarding in the field. We quantified foraging activity of males and estimated how their body condition is influenced by the duration of the caring period. Additionally, we conducted a one-year capture-mark-recapture study and estimated apparent survival probabilities of caring and non-caring males to assess potential survival costs of paternal care. Our results indicate that caring males forage less frequently than non-caring individuals (males and females) and that their body condition deteriorates over the course of the caring period. Thus, males willing to guard eggs may provide to females a fitness-enhancing gift of cost-free care of their offspring. Caring males, however, did not show lower survival probabilities when compared to both non-caring males and females. Reduction in mortality risks as a result of remaining stationary, combined with the benefits of improving egg survival, may have played an important and previously unsuspected role favoring the evolution of paternal care. Moreover, males exhibiting paternal care could also provide an honest signal of their quality as offspring defenders, and thus female preference for caring males could be responsible for maintaining the trait.

## Introduction

Trivers' [1] classical definition of parental investment postulates that even simple forms of parental care, such as egg-guarding, should include both benefits (enjoyed by the offspring) and costs (directly paid by parental individuals). The benefits of parental care to the offspring include improving micro-climatic conditions, such as reducing risk of dehydration and/or increasing egg aeration, protection against predators, parasitoid or fungal attack, as well as provisioning water or food to juveniles [2]. The costs paid by parental individuals are generally classified into three main categories [2]–[3]: (a) energetic costs, as a consequence of either reduced feeding opportunities or increased metabolic expense while caring for the offspring; (b) survival costs, as an ultimate consequence of starvation or increased susceptibility of the tending parent(s) to predators, parasites, and parasitoids; and (c) reproductive costs, involving loss of additional mating opportunities.

Parental care generally prevents foraging activities of parental individuals and can also be associated with expensive behaviors, such as providing offspring with food [2]. Therefore, maternal care is energetically costly for females, especially in the case of iteroparous species [4], since it reduces the available energy to produce additional eggs, negatively affecting females' future reproduction and fecundity (e.g., [5]–[9]). Among arthropods exhibiting exclusive paternal care, the available data are equivocal. Studies with giant water bugs (Belostomatinae), whose males carry egg pads attached to their backs, demonstrated that paternal behavior carries energetic costs for parental individuals by both decreasing their foraging efficiency and food intake [10], and by increasing their muscular activity while promoting water flow and oxygen diffusion through the eggs' membrane [11]–[12]. On the other hand, studies with the sea spider *Achelia simplissima* [13] and the assassin bug *Rhinocoris tristis* [9] showed that, besides differences in movement and activity patterns between caring and non-caring individuals, paternal care does not negatively affect foraging efficiency or weight loss, respectively.

Conflicting evidence is also the case for the survival costs of exclusive paternal care, for which empirical data are restricted to a few insect species. Observational data on the thrips *Hoplothrips karnyi* showed that caring males remain near communal egg masses, and the protection of these eggs against attacking conspecific males increases their mortality as a consequence of fighting injuries [14]. On the other hand, a laboratory study with the giant water bug *Belostoma flumineum* showed that the mean lifespan of males that had their egg pads removed was not different from either virgin or brooding males [15]. Only two studies using mark-recapture techniques to compare apparent survival probabilities between caring and non-caring males in the field have been conducted so far. For the giant water bug *Abedus breviceps*, males with eggs on their backs paid no survival costs [16], whereas for the assassin bug *R. tristis*, there was evidence of survival costs for caring males [9].

Finally, the reduction of mating opportunities for males during parental care is often pointed out as the most important cost of paternal care in endotherms because a trade-off is expected to exist between parental effort and mating effort [1], [3], [17]. Among many fishes and arthropods, however, eggs laid by different females can be guarded simultaneously, greatly reducing the promiscuity costs for caring males. In fact, observational and experimental evidence for these animal groups clearly show that providing paternal care and acquiring new mates are non-mutually exclusive activities [18]–[20]. Moreover, theoretical studies have suggested that female preferences for caring males have played an important role in the evolution of paternal care [18], [21]–[22]. Experimental evidence supporting this suggestion has already been reported for several fishes (e.g., [23]–[26]) and at least two arthropod species [9], [27].

**Figure 1:**
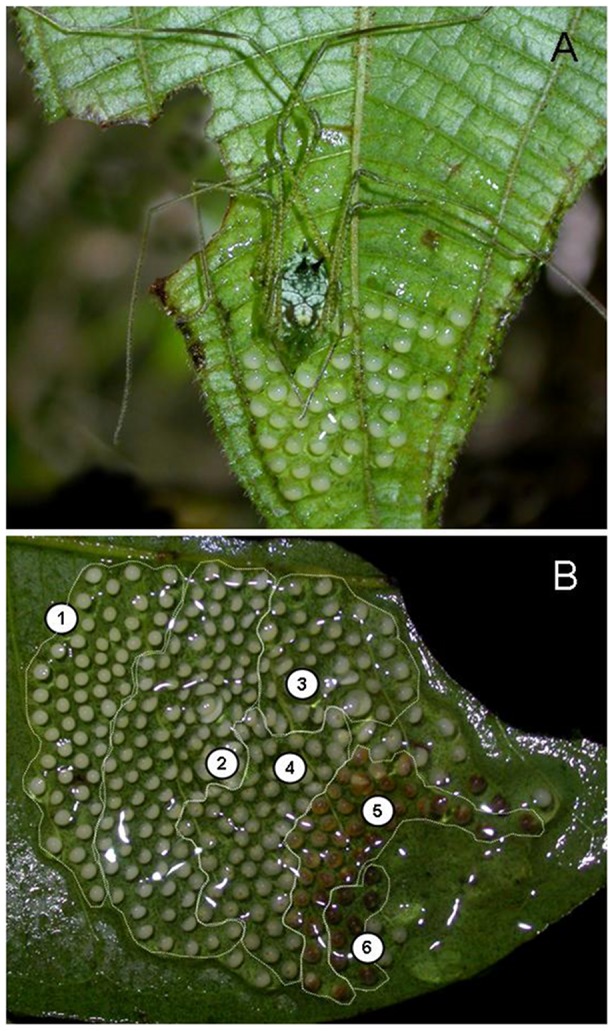
Paternal care in the harvestman *Iporangaia pustulosa*. (A) Male guarding eggs on the undersurface of a leaf. (B) Egg-batch composed of multiples clutches in different stages of embryonic development (see text for definition of each stage). Note that the eggs are covered by a thick mucus coat.

Although many theoretical models predict that the maintenance of parental care depends on the balance between costs and benefits of this behavior to the parents [2], [20], [22], empirical information among arthropods is restricted mostly to species exhibiting maternal care (e.g., [6]–[8], but see [9], [16]). In this paper, we used the Neotropical harvestman *Iporangaia pustulosa* (Arachnida: Opiliones) as a model organism to assess energetic and survival costs of male egg-guarding behavior under field conditions. *Iporangaia* females lay their eggs on the underside surface of leaves, secrete an abundant hygroscopic mucus coat on the clutch, and then abandon the eggs under males' protection [28]–[29] (Fig. 1A). During most part of the year, caring males remain on the eggs almost all the time [28], and their presence has a crucial protective role, given that unprotected eggs suffer intense predation in a few days [29]. Caring for the offspring and acquiring new mates are not mutually exclusive activities because males are able to sequentially copulate with several females and usually care for all their eggs simultaneously in a single multiple clutch [28] (Fig. 1B). Given that the clutches are acquired asynchronously by caring males, the total caring period may last up to four consecutive months [28], during which males are likely to experience both higher mortality probabilities and lower food intake when compared to non-caring individuals (males and females).

In this study, we evaluated energetic costs by quantifying foraging activity and estimating how body condition changes over the course of the caring period. Given that *Iporangaia* individuals feed mainly on dead arthropods, actively searching for food on the vegetation (G.S. Requena, unpublished data), we predicted that: (a) caring males would have fewer feeding opportunities when compared to non-caring individuals in the population, and (b) body condition would deteriorate over the course of the caring period. To evaluate mortality risk, we conducted a capture-mark-recapture study to estimate apparent survival probabilities and dissociate them from recapture probabilities [30]. Because parental care may increase the susceptibility of caring males to natural enemies, we predicted that their survival would be lower than that of non-caring males and females. Our study, entirely conducted in the field, provides a comprehensive understanding of the main costs paid by *Iporangaia* caring males. Moreover, our results challenge some widespread ideas on the costs of paternal care and bring insightful implications for the maintenance of paternal care in arthropods as a sexually selected trait.

## Methods

### Study Site

**Figure 2:**
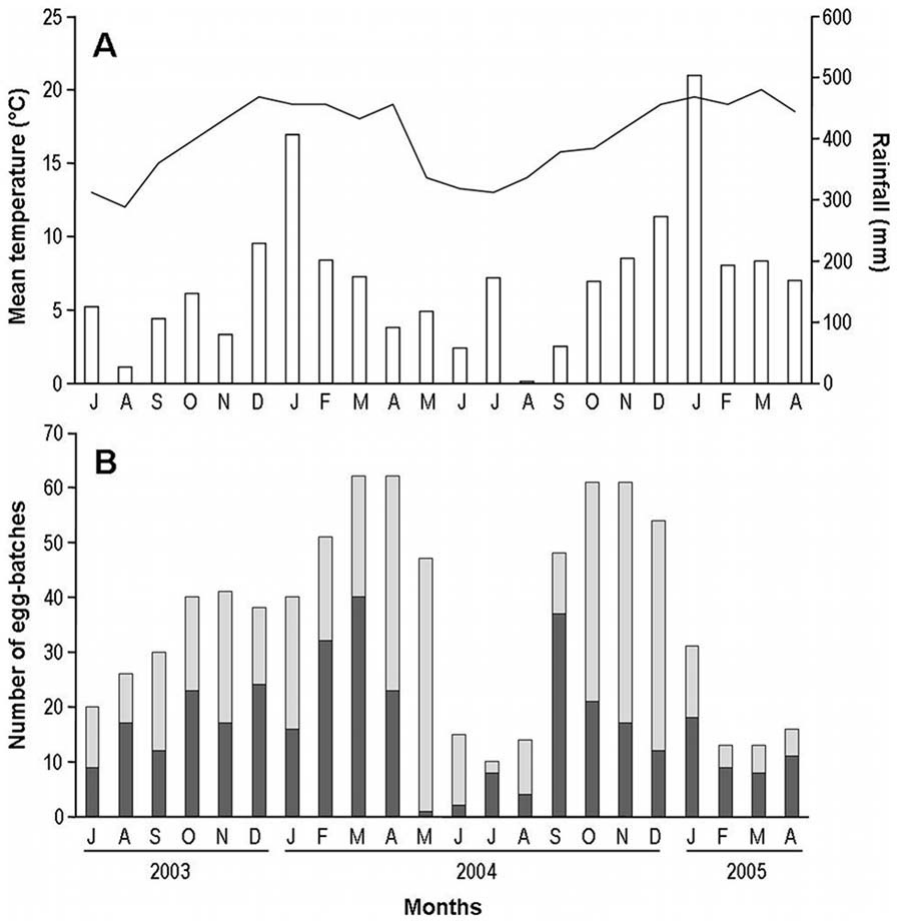
Climatic conditions and number of *Iporangaia* egg-batches observed in the sampled area. (A) The solid line represents temperature; black bars represent the rainfall during the dry-cold season; white bars represent the rainfall during the wet-warm season. (B) Dark-gray bars represent the number of new egg-batches found in each month (i.e., those that were not present in the transect in the previous months); light-gray bars represent old egg-batches (i.e., that were already present in the transect in the previous months).

We carried out this study in an Atlantic Forest fragment at Intervales State Park (24°14′S; 48°04′W), in the state of São Paulo, southeastern Brazil. The region has high precipitation levels, with an average annual rainfall of 2000 to 3000 mm/year, and mean annual temperature ranging from 12 to 20°C. There is a well-marked seasonality in the locality, with a wet and warm period from October to March, and a dry and cold period from April to September, when frosts are common (Fig. 2A). We collected our data along a stream nearly 5 m wide and flanked by abundant vegetation, which sometimes partially covers the stream bed. We established a 200 m transect along this stream and inspected the vegetation at a maximum distance of 1 m from the water in both margins. All procedures presented in the following sections were not conducted with endangered or protected species, and are in accordance with relevant national and international guidelines to ensure ethical appropriateness, for which we obtained all necessary permits from the authority responsible for Intervales State Park (COTEC-IF permit number: 40.625/05).

### Capture-mark-recapture

We conducted a one-year capture-mark-recapture study on our 200 m transect between August 2003 and July 2004. We inspected the vegetation flanking the stream three times a day (08.30–12.00 h, 14.30–18.00 h and 20.30–00.00 h) during four consecutive days per month. In each survey, we captured *Iporangaia* adults using an active searching method, recording their sex, their location along the transect (to the nearest 1 m), whether they were feeding and, for individuals captured for the first time, individually marking them with enamel color paint (following protocol described in [31]). After marking, we released the individuals at the same place where we had captured them. We classified males according to their parental state as caring (i.e., those that were guarding an egg-batch) or non-caring (i.e., those that were not guarding an egg-batch).

**Figure 3:**
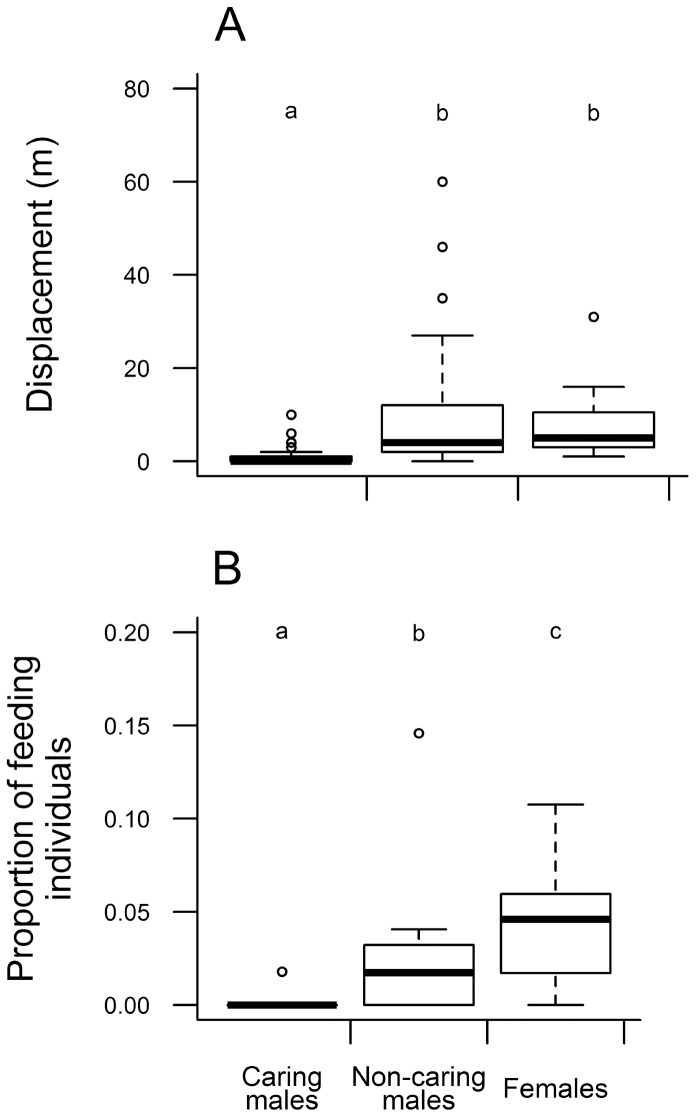
Movement pattern and feeding activity of *Iporangaia* adults. (A) Estimated distances that individuals in different parental states moved in consecutive capture occasions. (B) Observed proportion of individuals (females, caring and non-caring males) found in feeding activities in the field. Different letters represent differences among groups.

The capture-mark-recapture procedure also allowed us to estimate how males (caring and non-caring) and females moved along the transect. Considering only individuals that were recaptured at least five times and in at least two different months, we observed that both females (median = 5 m; range = 1–31 m) and non-caring males (median = 4 m; range = 1–60 m) moved similar distances among different sampling occasions and that it was always longer than the distances moved by caring males (median = 0 m, range = 0–10 m) (analysis of deviance: Δ deviance = 105.5, df = 1, *P*<0.001, Fig. 3A). This information is important to understand the results of the mark-recapture study because movement patterns may influence both recapture and mortality rates (see Discussion). Moreover, given that females and non-caring males have similar movement patterns and do not care for the offspring, we collectively classified them as ‘non-caring’ individuals in some analyses described below.

### Energetic Costs

To investigate energetic costs of paternal care in *Iporangaia*, we first quantified if there was any decrease in feeding activity to caring males when compared to other individuals in the population. *Iporangaia* individuals feed mostly on the vegetation and we recorded the monthly number of caring and non-caring individuals (females and males) observed in feeding activities during the capture-mark-recapture period and the total number of individuals recorded monthly in each category during the same period. These data are not without bias given that small food items are quickly consumed and recorded less frequently than large items. However, harvestmen are generalist and opportunistic consumers [32] so that we have no reason to suppose that caring and non-caring individuals exhibit preference for either small or large items. Thus, the number of individuals in each category that we found consuming food in the field seems to be a good proxy of their feeding activities.

We used a model selection approach based on the maximum likelihood method [33]–[34] to compare alternative generalized linear mixed models, representing different biological hypotheses. We built alternative models considering that the monthly proportion of feeding individuals was: (a) affected by neither sex nor parental state, (b) affected by sex (‘males’ vs. ‘females’), (c) affected by parental state (‘caring males’ vs. ‘non-caring males and females’), and (d) affected both individual categories (‘females’ vs. ‘caring’ vs. ‘non-caring males’). We built all models considering a binomial distribution of errors. Furthermore, given that we were not specifically interested in evaluating seasonal variation on foraging activity, but on the potential differences among categories, we used the sampling month as a random effect to control for such variation.

Then, we used the small sample size bias-corrected version of the Akaike Information Criterion (AIC*_c_*) to rank all models fitted to the data [34]. Then, we selected the model with the lowest AIC*_c_* value as the most parsimonious model describing the data. We also computed the difference in the AIC*_c_* value between the selected model and all other models in the ranking and the relative weight of all models. When this difference is larger than 2, there is strong support to conclude that the model selected is the best one among all candidate models [34]. We built, fit, and selected models using the packages ‘bbmle’ [35] and ‘lme4’ [36] in the software R 2.11.1 [37].

Our second approach to evaluate energetic costs of paternal care was to quantify how males' body condition changes over the course of the caring period. Between January and March 2009, we sampled 93 *Iporangaia* males in the field and, using an electronic caliper (precision of 0.01 mm) and an electronic scale (precision of 0.001 g), we took the following measurements from each individual: (a) dorsal scute length (*DSL*), (b) total body length (*TBL*), (c) body width (*BW*) at the widest portion of the opisthosoma, (d) body height (*BH*) at the highest portion of the opisthosoma, and (e) total body mass (*TBM*). The dorsal scute (or carapace) is a rigid structure that does not change in size with food acquisition and can be classified as a structural body size measure (*sensu* [38]). The last five opisthosomal tergites, on the other hand, are not fused, but rather connected by a highly elastic membrane, allowing body expansion after a meal.

Although body dimensions are presumably correlated with current nutrient storage and have been broadly used to estimate body condition, they actually have the disadvantage of being simultaneously correlated with structural body size [38]. To remove the effect of the latter, we used two different proxies to assess males' body condition (as suggested by [39]): (a) body volume (*V*) controlled by a structural body size measure (i.e. *DSL*), and (b) body mass (*TBM*) controlled by body volume (*V*). In our case, we estimated *V* as an approximation of the ellipsoid according to the following formula: 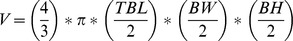.

Given that *Iporangaia* eggs change in size and coloration over the course of the embryonic development [28], we estimated the time males had already invested in paternal activities based on features of their egg-batches. During the same summer that we collected data on the body condition of males, we also photographed 20 egg-batches on a daily basis and used the temporal sequence of photos to estimate the duration of each stage of embryonic development, creating an egg development schedule that was used as a proxy for the caring period (Table 1, Fig. 1B). For non-caring males, we attributed the value zero to the duration of their caring period. For caring males, we considered the oldest eggs in their egg-batches as the time invested in paternal activities (Table 1). The first stage corresponds to recently laid eggs, which are completely white and have not yet absorbed water from the environment. Eggs in the second stage have the same color as those of the first stage, but are larger because of water absorption. In the second stage it is also possible to identify clearly the embryonic formation of legs. Eggs in the third stage are larger than those of the second stage and are generally opaque or milky, with the legs not so clearly identifiable. In the following three categories there is no more difference in egg size. Eggs in the fourth stage are similar in color to those of the third stage, but it is possible to clearly identify two black spots corresponding to the eyes of the embryo. Eggs in the fifth stage are dark yellow or brownish, which corresponds to the beginning of tegumentary pigmentation of the embryo. In the sixth and last stage, immediately before hatching, eggs are almost black (modified from [28]).

**Table 1:** Duration of each stage of embryonic development in Iporangaia eggs during the wet and warm season and the corresponding estimated caring period.

Stage	*n*	Duration range (days)	Cumulative duration (days)
1^st^	20	5–10	6.6
2^nd^	43	2–4	9.7
3^rd^	26	6–10	18.2
4^th^	10	5–8	24.5
5^th^	10	4–5	28.9
6^th^	7	2–4	32.0

NOTE. - *n* indicates the number of eggs from different oviposition events that were sampled to estimate the duration of each stage. ‘Cumulative duration’ is an estimation of how long it takes for recently laid eggs to complete specific embryonic development stages, calculated as the sum of the median observed duration of all previous stages.

Considering body volume as a proxy for body condition, we conducted the model selection procedure in two steps. First, to control for the effect of body size, we built models in which *DSL*: (a) does not affect *V*, (b) affects only the mean of *V*, (c) affects only the variance of *V*, and (d) affects both the mean and the variance of *V*. We used a linear function to model the influence on the mean parameter of *V* and a power function to model the variance parameter of *V* (as recommended by [40]). Using the best model selected in this first step, we then incorporated the effect of paternal care as: (a) the effect of caring period on the mean of *V* (controlled by body size), (b) caring period on the variance, (c) caring period on both parameters, (d) parental state on the mean, (e) parental state on the variance, and (f) parental state on both parameters. Using total body mass (*TBM*) as another proxy for body condition, we used the same two analytical steps described above to control for the effect of *V* and to evaluate the effect of paternal care on the mean and variance parameters of the *TBM* distribution. We conducted all these analyses and the model selection using the packages ‘bbmle’ [35] and ‘lme4’ [36] in the software R 2.11.1 [37], using AIC*_c_* to rank the models fitted to the data [34], as described above.

### Mortality Risk

To quantify the potential survival costs of male egg-guarding behavior, we estimated apparent survival (*Φ*) and recapture probabilities of females and males (caring and non-caring), using a statistical modeling approach [31] implemented in the software MARK [41]. We analyzed the data of all individuals together to be able to explicitly address models that consider the same *Φ* or among individuals of the same sex or individuals performing similar behaviors (parental state). Moreover, we pooled the capture-recapture data obtained during the three periods of the same day to generate a single sampling occasion per day. Therefore, our capture-recapture data set comprises 12 primary occasions (sampling months) and 46 secondary occasions (due to heavy rains, two months had only three days sampled). This so-called robust design model assumes that the population is open during the intervals between primary occasions, during which individuals may migrate, die or molt to the adult stage in the sampling area, but it is considered closed within each primary occasion because secondary occasions are so close together in time [42]. Therefore, it combines the advantages of closed capture models to estimate within primary occasions, at the *i^th^* month, with the advantages of the Cormack-Jolly-Seber live recapture model to estimate *ψ* between consecutive primary occasions, at the interval between the *i^th^* and the *(i+1)^th^* months (the model is described in details by [42]).

Male parental state is a varying condition in *Iporangaia* because caring males become non-caring males when nymphs hatch and disperse, while non-caring males may copulate and obtain a first clutch, thus becoming caring males. Therefore, we used multi-state models to estimate transition probabilities (*ψ*) between caring and non-caring states, at the interval between the *i^th^* and the *(i+1)^th^* months [43]–[45]. We used a ‘Huggins closed robust design multi-state model’, which does not include the abundance of individuals as a parameter of the model [42]. Furthermore, we also assumed the same probability to capture individuals for the first time and to recapture them within each primary occasion, and fixed *ψ* between males and females as zero.

We first assessed the fit of the global model to the capture-recapture history data. This global model considered that *Φ* and ***p*** were a function of time and individual categories (‘caring males’ vs. ‘non-caring males’ vs. ‘females’). Furthermore, the global model also considered that *ψ* between male states (‘caring’ vs. ‘non-caring’) was a function of time and parental state of males at the *i^th^* month. Our global model did not include interactions between time and individual categories (or parental state) because such a model did not reach convergence. We used the ratio of the model deviance by the model degrees of freedom, obtained by the goodness-of fit test for multi-state models performed in the software U-CARE [46], to estimate the overdispersion parameter of the global model (*ĉ*). The global model was considered to fit the data adequately if the estimated value of *ĉ* fell between 1 and 3, though the closer the value of *ĉ* is to 1, the better the fit of the model [41].

We compared the global model to three other general models incorporating different surrogates for time-dependent parameters. In the first model, we divided the study period into two seasons, corresponding to the wet-warm season (between October and March) and the dry-cold season (between April and September) (Fig. 2A). In the second and third models, we incorporated either temperature or rainfall as covariates representing time variation over the course of the study period, since both variables are strongly correlated with the reproductive activity of *Iporangaia* (Fig. 2B). For *Φ* and *ψ* estimates, we used the mean values of temperature and rainfall recorded for the days between two consecutive sampling occasions. For ***p*** estimates, we used the mean values of temperature and rainfall recorded during the four sampling days of each month. All additional models included interactions between time surrogates and individual categories (or parental state). We compared these four general models and selected the most parsimonious one using the small-sample size Akaike information criterion in the same way described in the *Energetic Costs* above, but corrected for overdispersion (QAIC_c_).

After the selection of the general model, we built models in the following way. First, we fixed the global structure for *Φ* and ***p*** as dependent on the additive effect between the time-related variable and individual categories, and built alternative models that considered *ψ* between caring and non-caring males as being: (a) constant and not affected by male state at the *i^th^* month, (b) affected by the selected time variable, (c) affected by male state at the *i^th^* month (‘caring’ vs. ‘non-caring’), and (d) affected by the additive effect of the selected time-related variable and the male state at the *i^th^* month. We also built two additional models in which (e) *ψ* from caring to non-caring state was constant, but *ψ* from non-caring to caring state was dependent of the selected time variable, and (f) *ψ* from non-caring to caring state was constant, but *ψ* from caring to non-caring state dependent of the selected time variable. We compared all alternative models and selected the best one using the QAIC_c_.

With the best selected structure for *ψ*, and with the structure for *Φ* still fixed as the additive effect between the time-related variable and individual categories, we built a new set of alternative models that considered ***p*** as being: (a) constant and not affected by individual categories, (b) affected by the selected time-related variable, (c) affected by individual categories, (d) affected by the additive effect of the time-related variable and individual categories, (e) constant for caring and non-caring males, but affected by the time-related variable for females, (f) constant for caring males and females, but affected by the time-related variable for non-caring males, (g) constant for non-caring males and females, but affected by the time-related variable for caring males, (h) affected by sex (‘all males together’ vs. ‘females’); (i) affected by the additive effect of the time-related variable and sex, (j) constant for males, but affected by the time-related variable for females, (k) constant for females, but affected by the time-related variable for males, (l) affected by parental state (‘non-caring males and females together’ vs. ‘caring males’), (m) affected by the additive effect of the time-related variable and parental state, (n) constant for caring individuals, but affected by the time-related variable for non-caring individuals, and (o) constant for non-caring individuals, but affected by the time-related variable for caring individuals, Finally, we built the same last 15 alternative models for *Φ* and repeated the model selection procedure.

We used the ‘step-down’ approach described above (and first presented by [30]) to avoid the comparison of all possible models in a single analysis, i.e., (4 structures for the general model)×(6 structures for *ψ*)×(15 structures for ***p***)×(15 structures for *Φ*) = 5,400 models, which would be a prohibitive, time consuming procedure and would greatly increase the possibility of spurious results [34], [47]. However, it is still not clear if the order in which the structure of parameters is fixed or modeled affects the convergence of different approaches to the same best selected model [30], [47]. In an attempt to avoid biased results due to our specific analytical implementation, we also performed the model selection procedure starting with a general model in which all parameters were considered constant. Both procedures converged to the same best supported model given the capture history data observed. Therefore, for the sake of simplicity, we will focus our results on the first step-down model selection procedure, in which we started with all parameters as dependent of the additive effect between the time-related variable and individual categories. Furthermore, since the estimates of ***p*** and *ψ* are not the main goal of our study, we will focus here on the results on estimates of *Φ*. The results of the second model selection procedure are presented in the Supporting Information S1 and the values of all additional probabilities included in the best supported model can be found in the Supporting Information S2.

## Results

### Energetic Costs

We found 501 males and 349 females of *Iporangaia*, recording a total of 3,503 captures and recaptures between August 2003 and July 2004. Of all males captured in the study area, 66.4% were recorded only in the non-parental state (*n* = 333), 12.4% were recorded only in the parental state (*n* = 62), and 21.2% were recorded in both parental states (*n* = 106). During this period, we observed 60 individuals feeding on the vegetation and most of them were recorded in the afternoon (14.30–18.00 h; 40%) and at night (20.30–00.00 h; 42%). From all individuals found while feeding, 35 were females, 24 were non-caring males, and only one was a caring male. Therefore, there was a clear effect of individual categories on feeding activity: the frequency of caring males feeding in the field was significantly lower than the frequency of non-caring males, and females were more often found in feeding activities than males in general (Table 2; Fig. 3B).

**Table 2:** Summary of the model selection statistics for the analysis that evaluated the monthly feeding activity of Iporangaia individuals between August 2003 and July 2004.

Predictor variables	AIC_c_	*K*	ΔAIC_c_	Weight
**Individual category (♂_C_ vs. ♂_NC_ vs. ♀)**	**53.4**	**4**	**0.0**	**0.926**
Parental state (♂_C_ vs. ♂_NC_+♀)	58.7	3	5.4	0.063
Sex (♂ vs. ♀)	62.3	3	9.0	0.011
No effects	76.5	2	23.2	<0.001

NOTE. Models are ranked by increasing order of their bias-corrected modified Akaike Information Criterion (AIC_c_). The best model is indicated in bold. ΔAIC_c_ is the difference between the AIC_c_ value of model *i* and the AIC_c_ value of the most parsimonious model; *K* is the number of estimable parameters in the model *i*; Weight is the Akaike weight of model *i*. The symbols ♂_C_, ♂_NC_ and ♀represent caring and non-caring males, and females, respectively.

**Figure 4:**
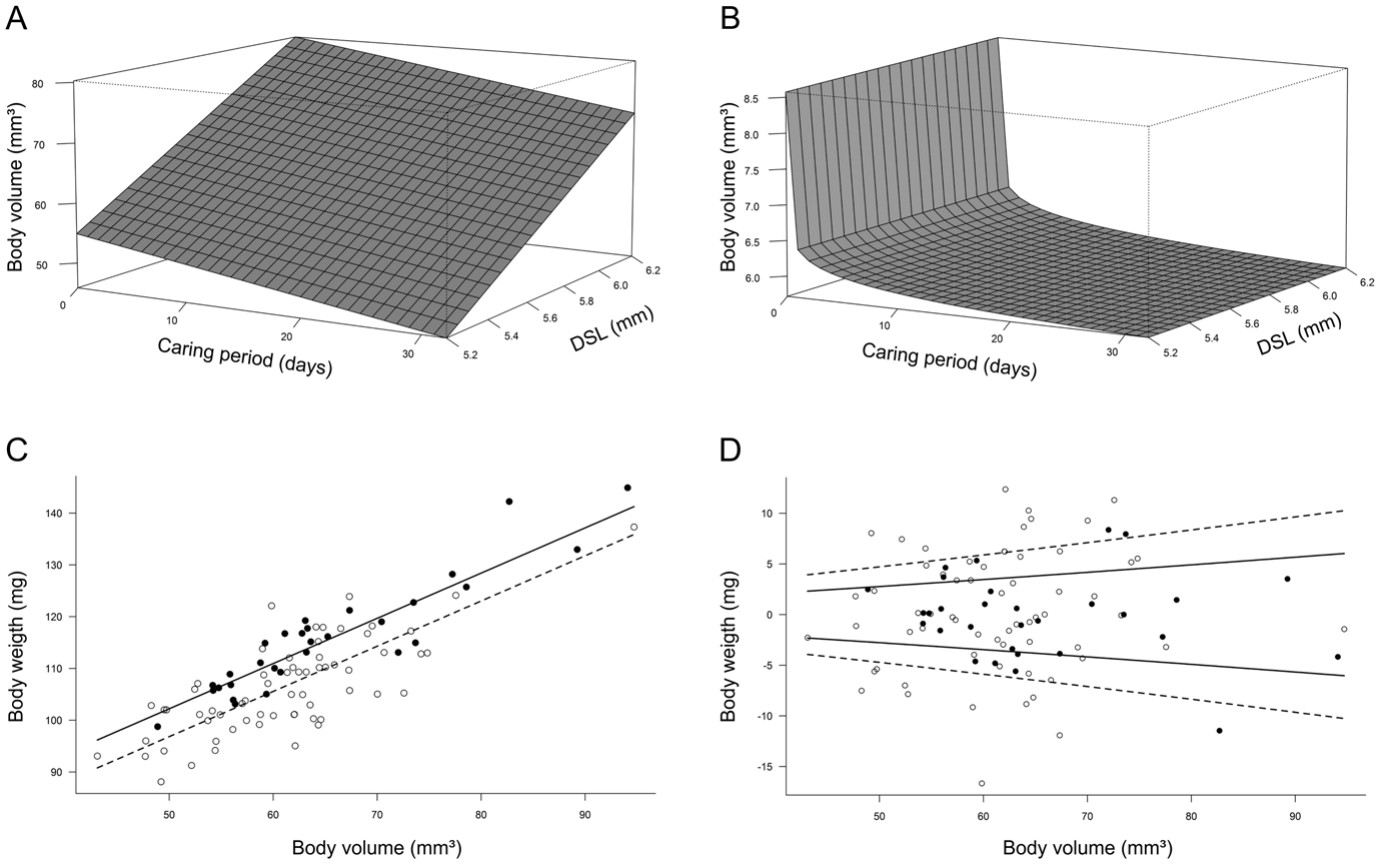
Energetic costs paid by *Iporangaia* caring males. Effect of caring period on the mean (A) and the variance (B) of males' body volume controlled by structural body size, i.e., dorsal scute length (*DSL*). Effect of parental state on the mean (C) and the variance (D) of males' weight controlled by body size, i.e., body volume (*V*). Filled circles and solid lines represent the predicted values for non-caring males, and open circles and dashed lines represent predicted values for caring males.

The best model to describe the relationship between males' structural body size (*DSL*) and males' body volume (*V*) was the one considering that *DSL* affects only the mean parameter of *V* distribution (Table 3). Then, using this model to control for the effect of body size, the most supported model taking into account the effect of paternal care considers that caring period negatively affects both the mean and the variance of *V* (Table 3). This means that males caring for eggs during longer periods have more homogenous and smaller body volumes than non-caring males or males that have just started to care (Fig. 4A–B). Therefore, we showed that our first proxy of body condition (body volume controlled by structural body size) decreases and is more homogeneous among *Iporangaia* males as caring period increases.

**Table 3:** Summary of the model selection statistics for the analysis that evaluated the relationship between paternal care and the mean and the variance of body condition proxies (controlled by structural body size) of Iporangaia males.

Models		AIC_c_	*K*	ΔAIC_c_	Weight
Mean	Variance				
*MALES' BODY VOLUME – V (STEP 1)*					
***DSL***	**-**	**651.6**	**3**	**0.0**	**0.752**
*DSL*	*DSL*	653.8	4	2.2	0.248
-	*DSL*	685.8	3	34.3	<0.001
-	*-*	686.5	2	34.9	<0.001
*MALES' BODY VOLUME – V (STEP 2)*					
***DSL*** **+caring period**	**Caring period**	**630.6**	**5**	**0.0**	**0.955**
*DSL*+caring period	-	636.8	4	6.2	0.044
*DSL*+parental state	Parental state	644.7	5	14.0	<0.001
*DSL*+parental state	-	646.2	4	15.5	<0.001
*DSL*	Caring period	647.4	4	16.8	<0.001
*DSL*	Parental state	648.5	4	17.8	<0.001
*DSL*	-	651.6	3	20.9	<0.001
*MALES' BODY MASS – TBM (STEP 1)*					
***V***	***V***	**604.7**	**4**	**0.0**	**0.525**
***V***	***-***	**604.9**	**3**	**0.2**	**0.475**
*-*	*V*	692.2	3	87.5	<0.001
*-*	*-*	706.6	2	101.9	<0.001
*MALES' BODY MASS – TBM (STEP 2)*					
***V*** **+parental state**	***V*** **+parental state**	**581.4**	**6**	**0.0**	**0.677**
*V*+parental state	Parental state	583.6	5	2.1	0.231
*V*+parental state	*-*	586.4	4	4.9	0.057
*V*+parental state	*V*	587.5	5	6.0	0.033
*V*+caring period	*V*+caring period	594.5	6	13.0	<0.001
*V*+caring period	Caring period	597.6	5	16.2	<0.001
*V*+caring period	*-*	599.3	4	17.9	<0.001
*V*+caring period	*V*	599.7	5	18.3	<0.001
*V*	*V*+parental state	603.9	5	22.5	<0.001
*V*	*V*+caring period	604.5	5	23.1	<0.001
*V*	*V*	604.7	4	23.3	<0.001
*V*	-	604.9	3	23.5	<0.001
*V*	Parental state	606.1	4	24.7	<0.001
*V*	Caring period	606.4	4	25.0	<0.001

NOTE. - Models are ranked by increasing order of their bias-corrected modified Akaike Information Criterion (AIC_c_). The best models in each stage are indicated in bold. ΔAIC_c_ is the difference between the AIC_c_ value of model *i* and the AIC_c_ value of the most parsimonious model; *K* is the number of estimable parameters in the model *I*; Weight is the Akaike weight of model *I*; *DSL* is the males' dorsal scute length (mm); *V* is the males' body volume (mm^3^); caring period is the estimated time males have already invested in parental activities (see text for methodological details); and parental state is the classification of males into the caring or non-caring categories.

Using total body mass (*TBM*) as another proxy for males' body condition, we found two equally plausible models to describe its relationship with the body size of males: considering the effect of males' body volume on the mean and variance of *TBM* distribution, and considering the effect of *V* only on the mean parameter of *TBM* (Table 3). Therefore, we used both model structures in the second analytical step, which revealed that the best supported model fitted to the observed data takes also into account the influence of parental state on the mean and variance of *TBM* (Table 3). For males of the same size, caring individuals were always lighter than non-caring males (Fig. 4C), although the variance in their body mass was higher (Fig. 4D). Therefore, our second proxy for body condition (body mass controlled by body volume) also negatively responded to variation in the caring period.

### Mortality Risk

**Figure 5:**
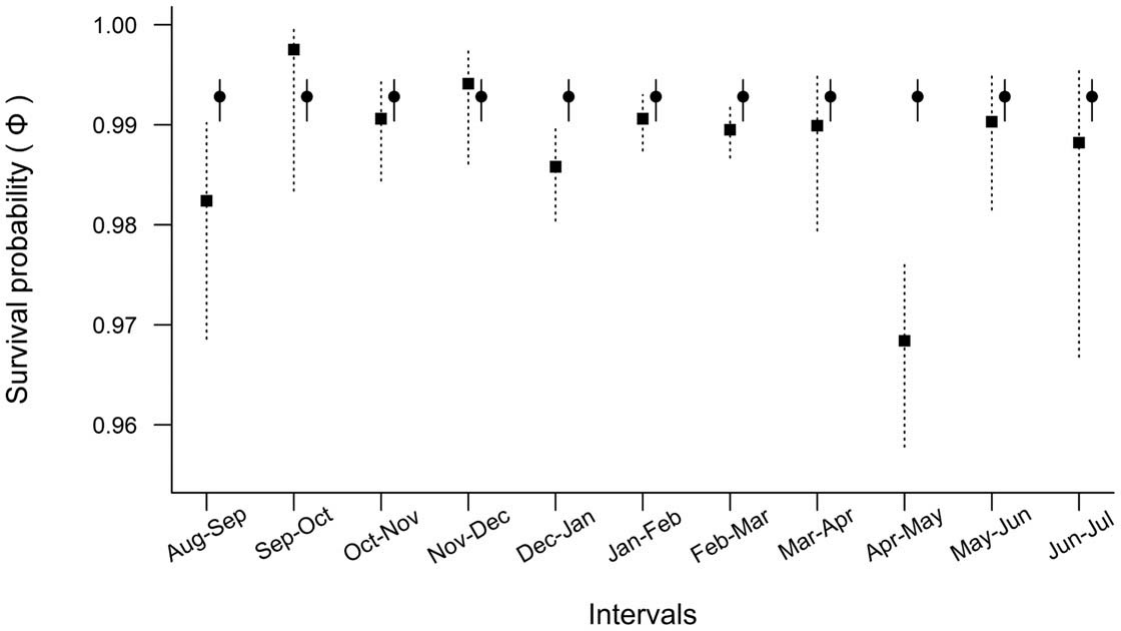
Apparent survival probability estimates for *Iporangaia* individuals according to their parental state. Vertical lines represent 95%CI of the monthly estimates, solid squares and dotted lines represent estimates for non-caring individuals (males and females), while solid circles and solid lines represent estimates for caring males.

The global model considering time dependence of all parameters fitted satisfactorily to the capture-recapture history data (goodness-of fit test for the JMV model: χ^2^ = 535.266, df = 485, *P* = 0.057), resulting in a *ĉ*-value of 1.1036. The global model including the additive effect of time and individual categories was by far the best supported by the data when compared to the models considering rainfall, temperature or seasons as time-related covariates (all had Δ QAIC_c_>50). The summary of the step-down model selection starting from the global model is shown in Table 4. At the end, the most supported model to explain the observed capture-recapture history data considered: (a) *ψ* as being influenced by the additive effect between time and the state of males; (b) ***p*** as being influenced by the additive effect between time and the individual categories in each sampled month; and (c) *Φ* as being influenced by parental state of individuals, with estimates for females and non-caring males depending on time, and estimates for caring males constant over the sampling period (Table 4). In this sense, non-caring males and females, which share similar behaviors, also showed similar apparent survival probabilities throughout the year. However, the best supported model revealed a general pattern that does not corroborate our initial hypothesis: caring males did not show lower survival than non-caring individuals. In fact, in almost all sampling months the survival estimates for parental males were higher than or at least similar to those of non-caring individuals (Fig. 5).

**Table 4:** Summary of the step-down model selection procedure for the capture-recapture analysis that investigated the influence of time, sex, parental state, and individual category on the transition probabilities between male parental states, as well as their influence on apparent survival and recapture probabilities of Iporangaia individuals between August 2003 and July 2004.

Parameter structure	QAIC_c_	*K*	ΔQAIC_c_	Weight
*FIRST STEP – TRANSITION PROBABILITY (ψ)*				
**Time+initial state**	**14,802.6**	**39**	**0.0**	**0.919**
Time (♂_NC_ to ♂_C_) & Constant (♂_C_ to ♂_NC_)	14,807.8	39	5.2	0.067
Time (♂_C_ to ♂_NC_) & Constant (♂_NC_ to ♂_C_)	14,811.0	39	8.4	0.013
Initial state	14,829.5	29	26.9	<0.001
Time	14,890.2	38	87.6	<0.001
Constant and not affected by initial state	14,897.4	28	94.8	<0.001
*SECOND STEP – RECAPTURE PROBABILITY (p)*				
**Time+individual category**	**14,802.6**	**39**	**0.0**	**0.692**
Time (♂_NC_ and ♀) & Constant (♂_C_)	14,805.1	38	2.5	0.191
Time+parental state	14,806.1	38	3.5	0.117
Time (♀) & Constant (♂_C_) & Constant (♂_NC_)	14,857.5	39	54.9	<0.001
Time (♂_NC_) & Constant (♂_C_) & Constant (♀)	14,910.0	39	107.4	<0.001
Time (♂_C_) & Constant (♂_NC_) & Constant (♀)	14,925.9	39	123.3	<0.001
Time (♂_C_) & Constant (♂_NC_ and ♀)	14,928.1	38	125.5	<0.001
Individual category	14,966.7	28	164.1	<0.001
Parental state	14,969.0	27	166.4	<0.001
Time (♀) & Constant (♂)	16,143.1	38	1,340.5	<0.001
Time+Sex	16,194.4	38	1,391.8	<0.001
Time (♂) & Constant (♀)	16,238.1	38	1,435.5	<0.001
Sex	16,254.3	27	1,451.7	<0.001
Time	16,459.3	37	1,656.7	<0.001
Constant and not affected by individual category	16,505.9	26	1,703.3	<0.001
*THIRD STEP – APPARENT SURVIVAL PROBABILITY (Φ)*				
**Time (♂_NC_ and ♀) & Constant (♂_C_)**	**14,799.6**	**38**	**0.0**	**0.781**
Time+individual category	14,802.6	39	3.0	0.181
Time+Sex	14,806.9	38	7.3	0.021
Time (♂_NC_) & Constant (♂_C_) & Constant (♀)	14,808.5	38	8.9	0.009
Time+parental state	14,809.4	38	9.8	0.006
Time (♂) & Constant (♀)	14,815.3	38	15.7	<0.001
Time (♀) & Constant (♂_C_) & Constant (♂_NC_)	14,815.5	39	15.9	<0.001
Individual category	14,815.6	29	16.0	<0.001
Sex	14,816.7	28	17.1	<0.001
Time (♀) & Constant (♂)	14,816.7	38	17.1	<0.001
Time	14,820.5	37	20.9	<0.001
Parental state	14,823.1	28	23.5	<0.001
Time (♂_C_) & Constant (♂_NC_) & Constant (♀)	14,824.9	39	25.3	<0.001
Constant and not affected by individual category	14,829.4	27	29.8	<0.001
Time (♂_C_) & Constant (♂_NC_ and ♀)	14,832.7	38	33.1	<0.001

NOTE. - Models are ranked by increasing order of their small-sample size and ĉ adjusted Akaike Information Criterion (QAIC_c_) for *ĉ* = 1.1036. The best models in each stage are indicated in bold. ‘ΔQAIC_c_’ is the difference between the QAIC_c_ value of model *i* and the QAIC_c_ value of the most parsimonious model; ‘*K* is the number of estimable parameters in the model *i*; ‘Weight’ is the Akaike weight of model *i*; ‘initial state’ represents the status individuals were classified at the *i^th^* month. ‘Individual category’ is a three-level classification factor for females (♀), caring (♂_C_) and non-caring males (♂_NC_); ‘parental state’ is a two-level a classification factor for caring and males and non-caring individuals (♀ and ♂_NC_); ‘sex’ is a two-level classification factor for females and males (♀ and ♂); ‘+’ represents the additive effects.

## Discussion

Our results indicate that parental behavior imposes energetic costs to *Iporangaia* caring males given that they feed less frequently than other individuals in the population and that their body condition deteriorates over the course of the caring period. However, the deterioration of body condition while guarding eggs does not seem to negatively affect the survival of caring males. Contrary to our initial hypothesis, survival estimates of males during the caring period were consistently higher than (or at least similar to) those obtained during the period in which they were not caring for the offspring. In the following sections, we will discuss these results in details and integrate them to explore the implications of our main findings for sexual selection and parental care theory.

### Energetic Costs

Both body volume (controlled by structural body size) and body mass (controlled by body volume) of caring males decreased as the time invested in egg-guarding increased. Given that the only parental activity exhibited by *Iporangaia* males is egg-guarding [28], the deterioration of body condition over the course of the caring period is probably the result of reduced food intake, rather than increased metabolic expense while caring for the offspring. Indeed, our field data indicate that caring males feed much less frequently, if at all, than caring males, a result similar to that obtained for another harvestman species with exclusive paternal care, *Magnispina neptunus* ( = *Pseudopucrolia* sp.) under laboratory conditions [27].

Most arthropod species exhibiting exclusive paternal care are predators or detritivorous [18], and males have developed strategies to maintain their body condition during the caring period. For example, sea spider males can carry egg-masses and seek food during brooding [13], while caring males of the assassin bug *R. tristis* usually perform filial cannibalism [48]. Instead of cannibalizing eggs, a rare behavior that we have observed only twice during more than 3,000 h of field observations, *Iporangaia* caring males may temporarily abandon the offspring to search for food [28]. However, given that body volume and mass of caring males clearly decreases over the course of the caring period, it seems that *Iporangaia*'s foraging behavior is not as efficient in maintaining caring males' body condition as the strategies reported for sea spiders [13] and assassin bugs [9] — probably because foraging is limited to a small area on the vegetation around the egg-batch where dead arthropods, an unpredictable food source, are likely to be scarce. Our results suggest, therefore, that the energetic costs of male care in this harvestman species are probably higher than the ones paid by the other two arthropod species in which the costs of paternal care have been measured.

Paternal care not only erodes *Iporangaia* males' body condition, but also results in a homogeneous body condition in caring individuals after a month of parental activities (Fig. 4B). Due to the increased attractiveness of males caring for recently laid egg-batches [49], they may copulate with several females at different moments, potentially prolonging the total caring period to more than three months [28] and, consequently, intensifying the cumulative energetic costs of caring. These costs associated with the extended period of care could explain why only a small fraction of males in the population (33.6%) were found caring for the offspring during one year of intensive sampling and why females avoid mating with males guarding egg-batches containing old eggs [49]. Poorly-fed males or males infested by larvae of phorid parasitoids [50] probably have limited endogenous energy reserves and are unable to pay the energetic costs of paternal care. Furthermore, depleted energy reserves may negatively affect the expected future quality of paternal care, measured as both the ability of males to protect the eggs against potential predators and the frequency and/or the duration of their temporary desertions to seek for food. These males, therefore, should be avoided by ovigerous females, just like has been reported for some fishes with paternal care (e.g. [51]–[52], but see [53]).

### Survival Costs

Although parental activities negatively affect body condition of *Iporangaia* males, egg-guarding per se does not seem to impose survival costs upon caring males. Here, we considered that the observed differences in apparent survival probability estimates among adults do represent real mortality, although the effects of permanent emigration and mortality are still confounded, even using a robust design model approach ([30], [44], but see detailed discussion in the Supporting Information S3). Two other field studies using a mark-recapture approach showed completely distinct patterns for arthropods. For the giant water bug *Abedus breviceps*, there was no difference in the apparent survival probability between males in caring and non-caring states [16]. The authors argued that males in both parental states are equally exposed to predators (mainly birds) during similar time periods, such as when water bugs go to the surface to take air. For the assassin bug *R. tristis*, on the other hand, the apparent survival probability of caring males was lower than that of non-guarding males [9]. Apparently, suppression of escape behavior in caring males, rather than their conspicuousness on the host plant, accounts for their lower survival. Due to the paucity of empirical evidence and the controversial results found by the available studies, the effect of paternal care on the survival of caring males certainly deserves further investigation in other arthropod groups. It seems clear, however, that the so-called effect of increased visibility of parental individuals to natural enemies [54] cannot account for all the empirical results reported so far.

Studies with Namib Desert beetles [55] and milkweed beetles [56] have reported that individuals that were more active during the reproductive period (males) were more frequently captured by ambush predators than sedentary individuals (females). In *Iporangaia*, females and non-caring males are constantly searching for mates and/or food inside home-range areas bigger than those of caring individuals, as seems evident by measurements of individual movements (Fig. 3A). Like the abovementioned beetles, it is plausible that females and non-caring males should be under stronger predation pressure than caring males, which remain close to their egg-batches for long periods. The natural predators recorded for Neotropical harvestmen in southeastern Brazil are frogs, mammals, insects, and spiders [50]. Half of the 18 known predatory species are active hunters (all vertebrate species, one assassin bug, and one ant species), and 66.6% of them forage primarily at the ground level. Thus, they are unlikely to prey on *Iporangaia*, which lives exclusively on the vegetation, mostly between 50 and 250 cm from the ground (G. S. Requena unpub. data). Conversely, the predators that adopt an ambush hunting strategy are spiders (nine different species) that catch their prey on the vegetation. Therefore, it is reasonable to assume that individuals of *Iporangaia* are more likely to be at risk of predation by ambush predators than by active hunters. Interestingly, the only predation event we witnessed in the field was by a corinnid spider that ambushed a female on the foliage (see Fig. 9.3 in [50]).

### Implications for Sexual Selection

Post-ovipositional maternal care in arthropods is a costly behavior because it reduces foraging opportunities for guarding females during long periods of care and, consequently, their lifetime fecundity (e.g. [7], [9], [57]). Given that the production of sperm and other seminal products generally requires fewer nutrients than does the production of eggs [58], care-related reductions in feeding activities are likely to be less costly for males than they are for females [1]. In species in which post-zygotic uniparental care is crucial for offspring survival, females leaving eggs under male protection are allowed to forage immediately after oviposition without sacrificing offspring survivorship. Here we demonstrated that, under field conditions, the foraging rate of *Iporangaia* females is also much higher than that of caring males, and similar to that of non-caring males. In this context, males willing to guard eggs may provide to females a fitness-enhancing gift of cost-free care of their offspring [17]. Under the male's perspective, reductions in the mortality risks as a result of remaining stationary, combined with the benefits of improving egg survival, may have selected originally for male parental care. Thus, contrary to current theoretical models, which assume that parental care increases male mortality [59], reductions in the mortality risks during the caring period may have played and important and previously unsuspected role favoring the evolution of paternal care.

Males exhibiting paternal care could also provide an honest signal of their quality as offspring defenders, and thus female preference for caring males could be responsible for maintaining the trait [18], [22]. Indeed, results from another field study with *Iporangaia* show that female choice seems to be influenced by the presence of eggs, and also by the age of the guarded offspring: caring males are preferred when they are guarding recently laid egg-batches and avoided when they are guarding old egg-batches in which nymphs have already hatched [49]. As we showed here, the longer the caring period, the worse the body condition of the male, which may negatively affect the quality of paternal care (see *Energetic Costs* above). Therefore, female rejection, mediated by poor male body condition and/or his low frequency of egg attendance, may prevent an indefinite increase in the number of eggs in an egg-batch. It is worth noticing, however, that the mucus coat secreted by *Iporangaia* females after oviposition may be viewed as a naturally-selected trait that confers protection to the eggs when starving males temporarily abandon their clutches to forage [28]–[29]. As a result, if the frequency of egg attendance decreases over the course of the caring period in response to the energetic costs imposed by prolonged male care, females are expected to invest more in the mucus coat when ovipositing in old egg-batches, whose males are probably food deprived. This is a testable hypothesis and *Iporangaia* offers the opportunity to investigate this putative conflict between sexes over the relative parental investment.

### Concluding remarks

Most models about life-history theory predict that parental care could evolve only when the benefits in terms of offspring survival outweigh the costs to the parents [2]. Furthermore, classic models usually assume that a trade-off does exist between parental and mating effort (see discussion in [20]). However, recent theoretical studies propose new benefits for males resulting from egg-guarding (as increased attractiveness and paternity for caring males), and point out that paternal care does not necessarily conflict with males' mating effort [18]–[20], [22]. Previous results from our research group indicate that paternal care in *Iporangaia* has an important protective role for the offspring, significantly decreasing egg predation [29], at the same time as it increases the attractiveness of caring males [49]. The findings we report here clearly show that food intake and body condition decline during the caring period, but this energetic cost does not reduce the survival of caring males. We conclude, therefore, that paternal care in this arthropod species incurs relatively low costs in relation to great benefits for caring males. Since the male egg-guarding behavior observed in *Iporangaia* is a simple form of parental assistance, further investigations in arthropod species in which males heavily invest in nest defense (e.g. [27], [60]) or carry large masses of eggs attached to their own body [11]–[12], would contribute to a more general understanding of the relationship among the intensity of paternal investment, the costs of caring, and the strength of sexual selection.

## Supporting Information

Table S1 **Summary of the first step-down model selection procedure for the capture-recapture analysis.**
(DOC)

Figure S1 **Transition probabilities, recapture probabilities, and population size estimates for the harvestman** ***Iporangaia pustulosa*** **.** (A) Transition probability estimates between male parental states. Solid diamonds represent estimates for the transition from non-caring to caring states, while filled diamonds represent estimates for the transition from caring to non-caring states. (B) Recapture probability and (C) population size estimates. Solid squares represent estimates for females; solid triangles represent estimates for non-caring males; and solid circles represent estimates for caring males. Vertical lines in all graphs represent 95%CI of the estimates in corresponding periods.
(DOC)

Figure S2 **Schematic representation of the study site.**
(DOC)
