# Supplementary material for: Paternal Care Decreases Foraging Activity and Body Condition, but Does Not Impose Survival Costs to Caring Males in a Neotropical Arachnid
Source: PLoS One. 2012 Oct 10;7(10):e46701. doi: 10.1371/journal.pone.0046701 (PMC3468633; doi:10.1371/journal.pone.0046701)
Supplement: Table S1 — Summary of the first step-down model selection procedure for the capture-recapture analysis. (DOC) [file pone.0046701.s001.doc]

**S.1 Results of the second model selection procedure for the capture-recapture data**

Table S1 shows the results of the second model selection procedure for the capture-recapture data. This model selection procedure converged to the same best supported model found in the first step-down procedure, in which we started with all parameters as dependent of the additive effect between the time-related variable and individual categories. The best supported model in both procedures consider: (a) *ψ* being influenced by the additive effect between time and the state of males; (b) *p* being influenced by the additive effect between time and the individual category in each sampled month; and (c) *ϕ* being influenced by parental state of individuals, with estimates for non-caring males and females depending on time and estimates for caring males constant over the sampled period.

**Table S1** Summary of the first step-down model selection procedure for the capture-recapture analysis

| **Parameter structure** | **QAIC_c_** | ***K*** | **∆QAIC_c_** | **Weight** |
| --- | --- | --- | --- | --- |
| First step – transition probability (*ψ)* | | | | |
| **Time + initial state** | **16,622.5** | **14** | **0.0** | **0.617** |
| **Time** (♂_C_ to ♂_NC_) & Constant (♂_NC_ to ♂_C_) | **16,623.4** | **14** | **0.9** | **0.381** |
| Initial state | 16,634.2 | 4 | 11.7 | 0.002 |
| Time | 16,648.8 | 13 | 26.3 | < 0.001 |
| Constant and not affected by initial state | 16,650.8 | 3 | 28.3 | < 0.001 |
| Second step – recapture probability (*p)* | | | | |
| **Time + individual category [M1]** | **14,829.4** | **27** | **0.0** | **0.835** |
| Time (♂_NC_ and ♀) & Constant (♂_C_) [M1] | 14,832.7 | 26 | 3.3 | 0.160 |
| Time + individual category [M2] | 14,840.8 | 27 | 11.4 | 0.003 |
| Time + parental state [M1] | 14,841.6 | 26 | 12.2 | 0.002 |
| Time (♂_NC_ and ♀) & Constant (♂_C_) [M2] | 14,845.0 | 26 | 15.6 | < 0.001 |
| Time + parental state [M2] | 14,851.8 | 26 | 22.4 | < 0.001 |
| Time (♀) & Constant (♂_C_) & Constant (♂_NC_) [M1] | 14,883.7 | 27 | 54.3 | < 0.001 |
| Time (♀) & Constant (♂_C_) & Constant (♂_NC_) [M2] | 14,892.6 | 27 | 63.2 | < 0.001 |
| Time (♂_NC_) & Constant (♂_C_) & Constant (♀) [M1] | 14,965.7 | 27 | 136.3 | < 0.001 |
| Time (♂_NC_) & Constant (♂_C_) & Constant (♀) [M2] | 14,977.5 | 27 | 148.1 | < 0.001 |
| Time (♂_C_) & Constant (♂_NC_) & Constant (♀) [M1] | 15,004.7 | 27 | 175.3 | < 0.001 |
| Time (♂_C_) & Constant (♂_NC_ and ♀) [M1] | 15,012.9 | 26 | 183.5 | < 0.001 |
| Time (♂_C_) & Constant (♂_NC_) & Constant (♀) [M2] | 15,013.6 | 27 | 184.2 | < 0.001 |
| Time (♂_NC_) & Constant (♂_NC_ and ♀) [M2] | 15,022.0 | 26 | 192.6 | < 0.001 |
| Individual category [M1] | 15,045.8 | 16 | 216.4 | < 0.001 |
| Parental state [M1] | 15,054.1 | 15 | 224.7 | < 0.001 |
| Individual category [M2] | 15,055.0 | 16 | 225.6 | < 0.001 |
| Parental state [M2] | 15,063.3 | 15 | 233.9 | < 0.001 |
| Time (♀) & Constant (♂) [M1] | 16,188.4 | 26 | 1,359.0 | < 0.001 |
| Time (♀) & Constant (♂) [M2] | 16,189.4 | 26 | 1,360.0 | < 0.001 |
| Time + Sex [M1] | 16,254.0 | 26 | 1,424.6 | < 0.001 |
| cont. |  |  |  |  |
| **Parameter structure** | **QAIC_c_** | ***K*** | **∆QAIC_c_** | **Weight** |
| Second step (cont.) – recapture probability (*p)* |  |  |  |  |
| Time + Sex [M2] | 16,255.0 | 26 | 1,425.6 | < 0.001 |
| Time (♂) & Constant (♀) [M1] | 16,322.1 | 26 | 1,492.7 | < 0.001 |
| Time (♂) & Constant (♀) [M2] | 16,323.1 | 26 | 1,493.7 | < 0.001 |
| Sex [M1] | 16,345.3 | 15 | 1,515.9 | < 0.001 |
| Sex [M2] | 16,346.3 | 15 | 1,516.9 | < 0.001 |
| Time [M1] | 16,558.4 | 25 | 1,729.0 | < 0.001 |
| Time [M2] | 16,559.3 | 25 | 1,729.9 | < 0.001 |
| Constant and not affected by individual category [M1] | 16,622.5 | 14 | 1,793.1 | < 0.001 |
| Constant and not affected by individual category [M2] | 16,623.4 | 14 | 1,794.0 | < 0.001 |
| Third step – apparent survival probability (*ϕ)* | | | | |
| **Time (♂_NC_ and ♀) & Constant (♂_C_)** | **14,799.6** | **38** | **0.0** | **0.781** |
| Time + individual category | 14,802.6 | 39 | 3.0 | 0.181 |
| Time + Sex | 14,806.9 | 38 | 7.3 | 0.021 |
| Time (♂_NC_) & Constant (♂_C_) & Constant (♀) | 14,808.5 | 38 | 8.9 | 0.009 |
| Time + parental state | 14,809.4 | 38 | 9.8 | 0.006 |
| Time (♂) & Constant (♀) | 14,815.3 | 38 | 15.7 | < 0.001 |
| Time (♀) & Constant (♂_C_) & Constant (♂_NC_) | 14,815.5 | 39 | 15.9 | < 0.001 |
| Individual category | 14,815.6 | 29 | 16.0 | < 0.001 |
| Sex | 14,816.7 | 28 | 17.1 | < 0.001 |
| Time (♀) & Constant (♂) | 14,816.7 | 38 | 17.1 | < 0.001 |
| Time | 14,820.5 | 37 | 20.9 | < 0.001 |
| Parental state | 14,823.1 | 28 | 23.5 | < 0.001 |
| Time (♂_C_) & Constant (♂_NC_) & Constant (♀) | 14,824.9 | 39 | 25.3 | < 0.001 |
| Constant and not affected by individual category | 14,829.4 | 27 | 29.8 | < 0.001 |
| Time (♂_C_) & Constant (♂_NC_ and ♀) | 14,832.7 | 38 | 33.1 | < 0.001 |

NOTE. - Models are ranked by increasing order of their small-sample size and ĉ adjusted Akaike Information Criterion (QAIC_c_) for ĉ = 1.105. The best models in each stage are indicated in bold. '∆QAIC_c_' is the difference between the QAIC_c_ value of model *i* and the QAIC_c_ value of the most parsimonious model; '*K* is the number of estimable parameters in the model *i; '*Weight' is the Akaike weight of model *i;* 'initial state' represents the status individuals were classified at the *i^th^* month. ‘Individual category’ is a three-level classification factor for females (♀), caring (♂_C_) and non-caring males (♂_NC_); ‘parental state’ is a two-level a classification factor for caring and males and non-caring individuals (♀ and ♂_NC_); 'sex' is a two-level classification factor for females and males (♀ and ♂); '+' represents the additive effects. Since two models were equally plausible during the first step, 'M1' and 'M2' represent the specific parameter structure of ψ between parental states during the second step of the model selection procedure, given by the best and the second best models, respectively.
