# Supplementary material for: Paternal Care Decreases Foraging Activity and Body Condition, but Does Not Impose Survival Costs to Caring Males in a Neotropical Arachnid
Source: PLoS One. 2012 Oct 10;7(10):e46701. doi: 10.1371/journal.pone.0046701 (PMC3468633; doi:10.1371/journal.pone.0046701)
Supplement: Figure S1 — Transition probabilities, recapture probabilities, and population size estimates for the harvestman Iporangaia pustulosa . (A) Transition probability estimates between male parental states. Solid diamonds represent estimates for the transition from non-caring to caring states, while filled diamonds represent estimates for the transition from caring to non-caring states. (B) Recapture probability and (C) population size estimates. Solid squares represent estimates for females; solid triangles represent estimates for non-caring males; and solid circles represent estimates for caring males. Vertical lines in all graphs represent 95%CI of the estimates in corresponding periods. (DOC) [file pone.0046701.s002.doc]

**S.2 Supplemental parameter estimates of the best supported model for the capture-recapture data**

Although the estimates of the transition probabilities between male parental states fluctuated over the sampling period, the transition estimates from non-caring to caring states were always lower than the corresponding transition estimates from caring to non-caring states (Fig. S1-A). Therefore, at any time, it is less likely than a non-caring male acquire a first clutch and become a caring male than the converse, probably because females prefer to lay eggs with caring males [28]. Regarding to the estimates of recapture probabilities, they showed an overall increase between January and March 2004 (Fig. S1-B), which coincides with the increase in the reproductive activity registered for that year (Fig. 2B). Furthermore, recapture estimates for caring males were between three and six times higher than estimates for non-caring males during the whole sampling period. Recapture estimates for non-caring males, on the other hand, were just a little higher than estimates for females. This pattern was probably generated due to behavioral differences related to movement patterns and parental care. While caring males usually stay close to their egg-batches, non-caring individuals (males and females) are actively searching for mates, food or shelters on the vegetation (Fig. 3A).

**
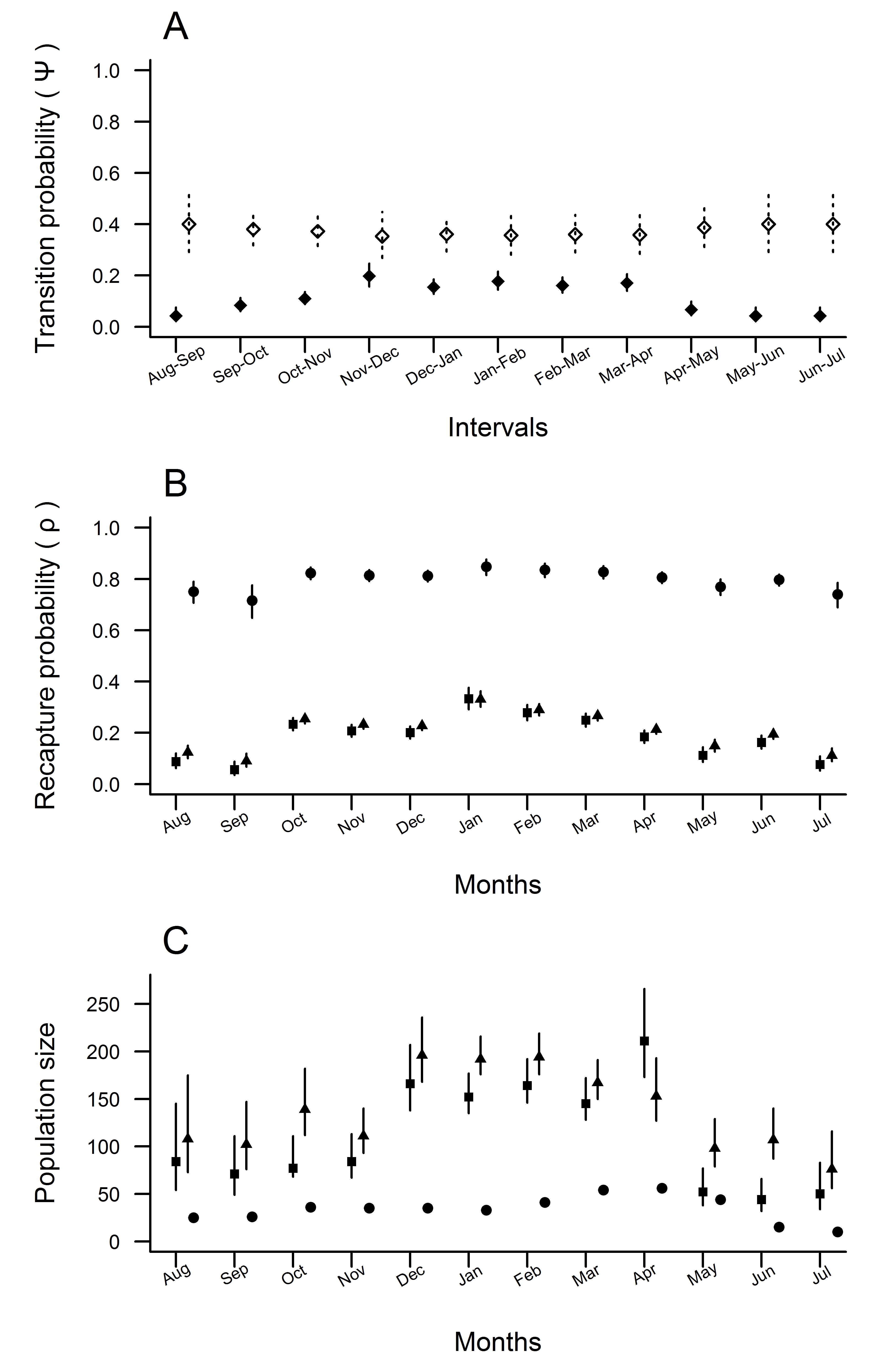

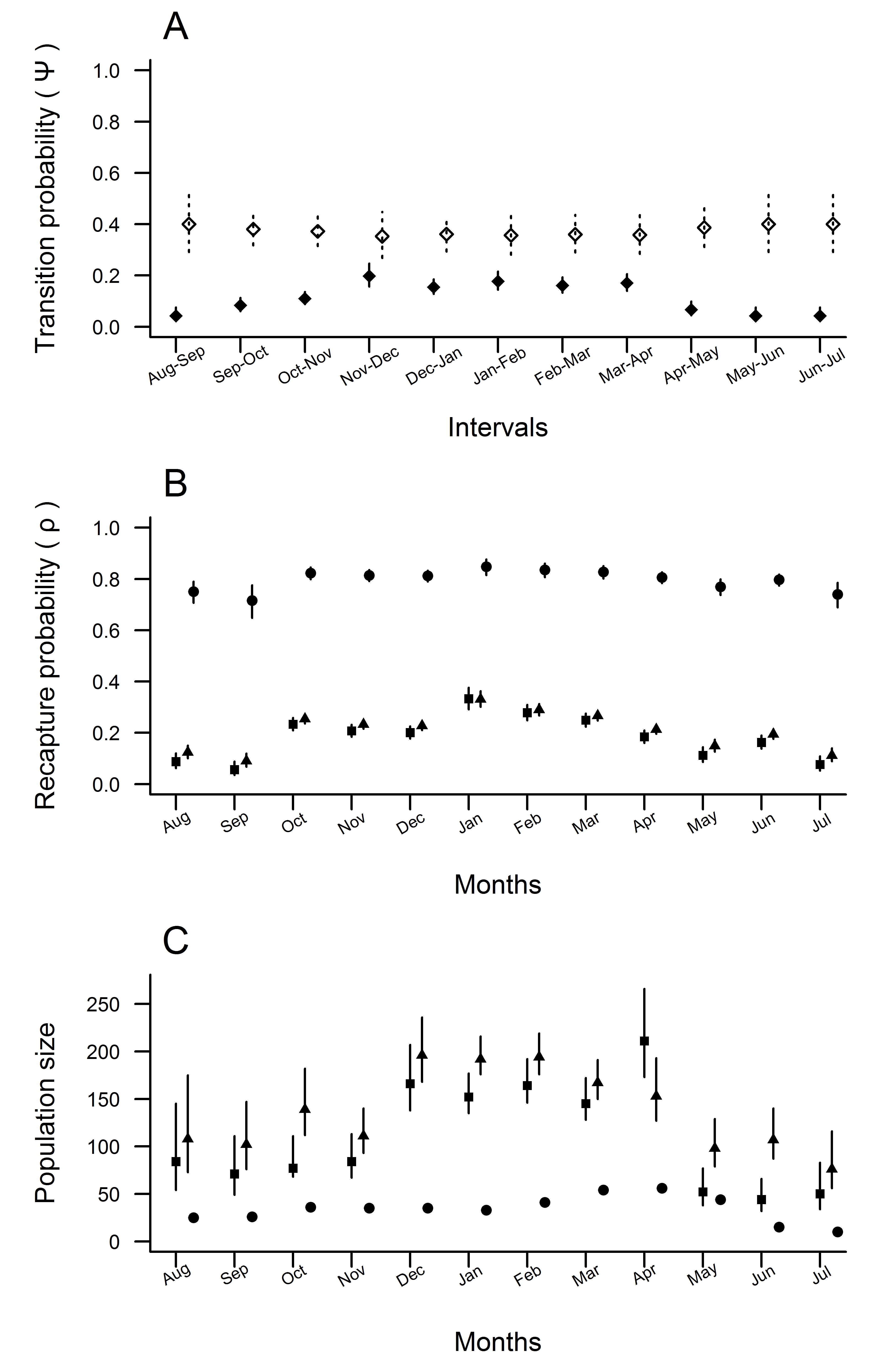
**

**Figure S1: Transition probabilities, recapture probabilities, and population size estimates for the harvestman *Iporangaia pustulosa*.** (A) Transition probability estimates between male parental states. Solid diamonds represent estimates for the transition from non-caring to caring states, while filled diamonds represent estimates for the transition from caring to non-caring states. (B) Recapture probability and (C) population size estimates. Solid squares represent estimates for females; solid triangles represent estimates for non-caring males; and solid circles represent estimates for caring males. Vertical lines in all graphs represent 95%CI of the estimates in corresponding periods.
