# Supplementary material for: Paternal Care Decreases Foraging Activity and Body Condition, but Does Not Impose Survival Costs to Caring Males in a Neotropical Arachnid
Source: PLoS One. 2012 Oct 10;7(10):e46701. doi: 10.1371/journal.pone.0046701 (PMC3468633; doi:10.1371/journal.pone.0046701)

**S.3 Schematic representation of the study site**

The beginning of our 200 m transect was located just after a 15 m high waterfall. We have never found any *Iporangaia* adult in the stream before this waterfall, and thus we suppose that it represents the upstream limit of the studied population. Along the first 250 m after the waterfall, the stream is nearly 5 m wide and the streambed is almost completely covered by the canopy formed by the marginal vegetation. After that, the stream becomes wider and the canopy formed by the marginal vegetation does not cover the streambed. *Iporangaia* individuals are found almost exclusively in shadowed areas of the stream (marked in gray) and the beginning of sunny area represents the downstream limit of the studied population. At one side of the stream, there is a steep riverbank (represented by the brown rectangle) where we have never found any egg-batch. Even adults are rarely found on the marginal vegetation close to this riverbank. At the other side, there is a parallel track 1 to 3 m from the stream, where we also searched for *Iporangaia* individuals during our fieldwork. Although we have found few egg-batches and adults on the vegetation at the margins of this track, most of them were close to stream. Moreover, we have never found adults in the forested area close to stream, suggesting that movements away from the stream are relatively rare. Therefore, our transect (inside the red dashed rectangle) is likely to include most part of the population, and even if adults migrate to other areas, it constitutes a minor effect to the estimates of survival probabilities.


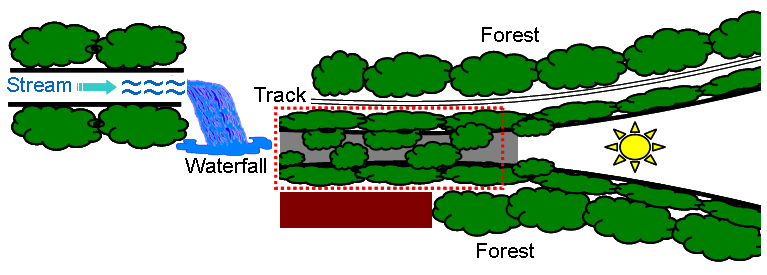

Supplement: Figure S2 — Schematic representation of the study site. (DOC) [file pone.0046701.s003.doc]
